# Activation of SIRT1 by Resveratrol requires lamin A

**DOI:** 10.18632/aging.100532

**Published:** 2013-02-21

**Authors:** Baohua Liu, Zhongjun Zhou

**Affiliations:** ^1^ Department of Biochemistry, Li Ka Shing Faculty of Medicine, The University of Hong Kong, Hong Kong; ^2^ Shenzhen Institute of Research and Innovation, The University of Hong Kong, Hong Kong

Resveratrol, a compound enriched in red grape skin, has been reported to increase lifespan in yeast, worms and flies and enhance healthspan in rodents. Beneficial effects of resveratrol have been reported in aging-related cataracts, bone loss, neurodegeneration, obesity and diabetes. Resveratrol induces multiple genes expression mimicking caloric restriction (CR), which is the most conserved longevity-promoting manipulation across species [1]. Resveratrol intake conferred metabolic changes similar as CR in obese individuals [2]. Short-term consumption of resveratrol achieved a similar effect of CR [3]. In 2004, David Sinclair's group from Harvard Medical School in Boston identified resveratrol for the first time as a “direct” activator of SIRT1 using a fluorophore-conjugated synthetic peptide as targets [4]. SIRT1 is a NAD^+^-dependent protein deacetylase and regulates various metabolic pathways. Loss of SIRT1 abolishes many beneficial effects of CR, while transgenic mice with additional copies of *SIRT1* show phenotypes resembling CR [5].

**Figure:**
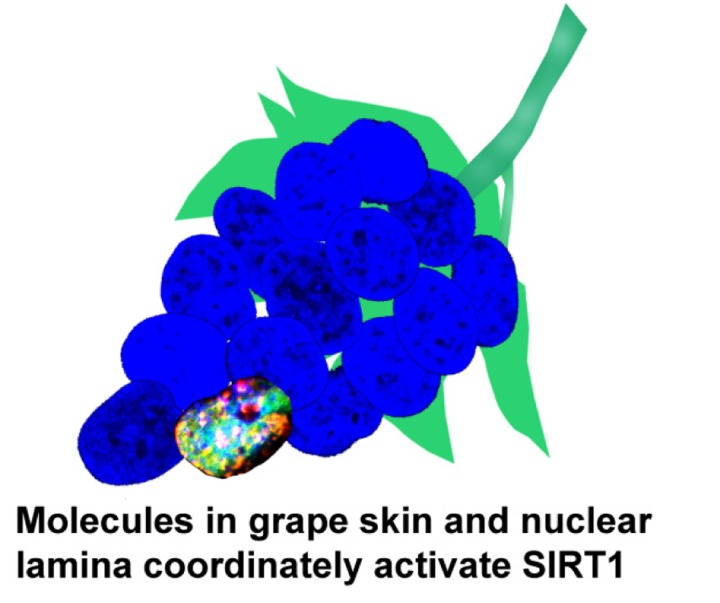


Over the past several years, CR-mimicking properties of resveratrol and SIRT1 have attracted considerable efforts in searching for resveratrol mimics. Based on the same screening strategy, researchers in Sirtris Pharmaceuticals found more than 5,000 SIRT1-activating compounds (STACs). One of the most characterized is SRT1720, which exhibits more than 1000-fold increase in SIRT1-activating potential compared with resveratrol and elicits similar CR-mimicking beneficial effects as resveratrol does [6]. However, in 2005, two independent groups reported that resveratrol only specifically enhances SIRT1 activity towards the fluorophore-conjugated rather than the unmodified synthetic peptide. This notion was later confirmed by other independent groups, showing that resveratrol and SRT1720 do not confer any SIRT1 activation towards its full-length native target proteins, including p53 and PGC-1α [7]. Although whether and how resveratrol activates SIRT1 remain unclear, many of its *in vivo* benefits have been shown to be dependent on SIRT1.

In the current issue of **Cell Metabolism** (Volume 16, Issue 6, 738-750, 5 December 2012), we reported thatlamin A directly binds to SIRT1 and serves as an endogenous activator of SIRT1. Resveratrol treatment activates SIRT1 *in vivo*, ameliorates progeroid features and extends lifespan in a Hutchinson-Gilford progeria syndrome (HGPS) mouse model, Zmpste24 null mice. Lamin A, encoded by the *LMNA* gene, is a major component of the nuclear lamina and nuclear matrix, a filamentous nucleoskeleton critical for maintenance of nuclear structure. In HGPS cells where substantial amount of lamin A is replaced with progerin [8], SIRT1 deacetylase activity was significantly reduced due to decreased association of SIRT1 with nuclear matrix. *In vivo* and *in vitro* experiments revealed that the binding of lamin A to SIRT1 protein is critical to SIRT1 activation. As only lamin A but not lamin C interacts with SIRT1, the C-terminal specific domain of lamin A is most likely responsible for the interaction; and the farnesylated carboxyl tail in progerin and prelamin A might interfere with the interaction as well as the activation of SIRT1. Although how resveratrol activates SIRT1 is still unclear, it is likely that the binding of lamin A with SIRT1 has an allosteric effect on SIRT1 conformation which exposes active site of SIRT1 deacetylase to it native substrates. In an effort to further investigate whether lamin A serves as a co-factor of resveratrol in the activation of SIRT1, we found that resveratrol alone does not activate SIRT1 towards its native target, e.g. full-length acetyl p53. Instead, resveratrol enhances the binding of SIRT1 to lamin A and thus increases SIRT1 deacetylase activity. One possible explanation is that resveratrol might modulate the SIRT1 conformation, thus increasing the binding of SIRT1 to lamin A and enhancing its deacetylase activity. An alternate explanation could be that resveratrol bridges new interacting domains of lamin A and SIRT1. In any case lamin A might serve as an allosteric effector of SIRT1. Given that the C-terminal 80 amino acids of lamin A exhibits higher potential in activating SIRT1, it is also possible that the remaining part of lamin A might impose a conformational barrier for SIRT1 activation, and resveratrol might lead to allosteric changes in the lamin A-SIRT1 complex and thus further activate SIRT1. Nevertheless, the present finding provides direct evidence that resveratrol works through Lamin A in activating SIRT1 and suggests a therapeutic strategy based on SIRT1 pathway for HGPS. Also provided is a screening strategy for SIRT1-activating/inhibiting compounds based on the interaction between lamin A and SIRT1 and SIRT1-activating property of lamin A. Since the increase in SIRT1 deacetylase activity also confers beneficial effects on various mouse models mimicking human metabolic or degenerative diseases, such as obesity, diabetes and Alzheimer Diseases, SIRT1-activating compounds could benefit human patients suffering from various metabolic and aging-related degenerative diseases. On the other hand, SIRT1 protein is found upregulated in various human cancers and SIRT1-inhibiting compounds can be used to treat human malignancies.
